# Changes in Chemical and Thermal Properties of Bamboo after Delignification Treatment

**DOI:** 10.3390/polym14132573

**Published:** 2022-06-24

**Authors:** Huiling Yu, Chengsheng Gui, Yaohui Ji, Xiaoyan Li, Fei Rao, Weiwei Huan, Luming Li

**Affiliations:** 1 College of Engineering, Yantai Nanshan University, Yantai 265713, China; yhl053558@163.com; 2 Zhejiang Shenghua Yunfeng New Material Co., Ltd., Huzhou 313200, China; gcs19882006@126.com; 3 Research Institute of Wood Industry, Chinese Academy of Forestry, Beijing 100091, China; jiyaohui1994@gmail.com; 4 China National Bamboo Research Center, Department of Efficient Utilization of Bamboo and Wood, Wenyi Road 310, Hangzhou 310012, China; xiaoyanli1995@sina.com; 5 School of Art and Design, Zhejiang Sci-Tech University, Hangzhou 310018, China; raofei@zstu.edu.cn; 6 College of Chemistry and Materials Engineering, Zhejiang A&F University, Hangzhou 311300, China; 20130874@zafu.edu.cn

**Keywords:** chemical change, thermal property, bamboo, delignification, pyrolysis, bamboo microstructure

## Abstract

Bamboo delignification is a common method for studying its functional value-added applications. In this study, bamboo samples were delignified by treatment with sodium chlorite. The effects of this treatment on the bamboo’s microstructure, surface chemical composition, and pyrolysis behaviour were evaluated. Field-emission scanning electron microscopy (FE-SEM), Fourier-transform infrared (FTIR) spectroscopy, X-ray photoelectron spectroscopy (XPS), and X-ray diffraction (XRD) were conducted to evaluate these parameters. The FTIR results demonstrated that the lignin peak decreased or disappeared, and some hemicellulose peaks decreased, indicating that sodium chlorite treatment effectively removed lignin and partly decomposed hemicellulose, although cellulose was less affected. The XPS results showed that, after treatment, the oxygen-to-carbon atomic ratio of delignified bamboo increased from 0.34 to 0.45, indicating a lack of lignin. XRD revealed increased crystallinity in delignified bamboo. Further pyrolysis analysis of treated and untreated bamboo showed that, although the pyrolysis stage of the delignified bamboo did not change, the maximum thermal degradation rate (R_max_) and its corresponding temperature (from 353.78 to 315.62 °C) decreased significantly, indicating that the pyrolysis intensity of the bamboo was weakened after delignification. Overall, this study showed that delignified bamboo develops loose surfaces, increased pores, and noticeable fibres, indicating that alkali-treated bamboo has promising application potential due to its novel and specific functionalities.

## 1. Introduction

Global ecological deterioration has shifted researchers’ focus onto natural materials, and issues such as environmental friendliness and recyclability are becoming increasingly important in the development of new materials. Consequently, cellulose, which is the most abundant biopolymer on Earth with these properties, has been widely used as a source of raw materials. Natural fibres in wood and bamboo materials are abundant, biodegradable, and eco-friendly resources. Hence, they are considered high-quality alternatives [1]. Consequently, these materials have become research hotspots in recent years. Several functional studies have recently been conducted on various wood and bamboo fibre materials, such as transparent wood, aesthetic wood, flame retardant fibre materials, oil–water separation sponges, supercapacitor electrodes, and bioplastics [2,3,4,5,6,7]. Naturally occurring lignocellulosic materials, such as wood and bamboo, are porous materials. However, to further increase wood’s porosity and susceptibility to functionalization, the most common strategy is delignification [8], which is the first and key step in studying the functionalization of lignocellulosic materials. As a result of delignification, nanopores in the cell wall structure become exposed. Additionally, delignification affects the chemical composition of wood, as it removes most of the lignin and part of the hemicellulose and facilitates the insertion of polymer and inorganic materials, thereby improving the wood’s mechanical properties, hydrophobicity, magnetism, insulation, or transparent properties [9,10]. Previous studies obtained cellulose scaffolds using delignification treatment, which helped to expand the range of application of cellulose. Therefore, delignification treatment as a method of extracting cellulose fibres from wood and bamboo has recently garnered substantial interest. To date, several pre-treatment methods, including physico-chemical [11], chemical [12,13], and biological [14,15] methods, have been investigated to remove lignin and separate cellulose in wood and bamboo. The chemical method primarily involves acid or alkali solution impregnation treatment, whereas the physico-chemical method mainly uses acid or alkali solution steam treatment. Although physico-chemical methods improve the efficiency of delignification, they are associated with high energy consumption and cost. The biological method uses fungi for biodegradation, which is a green preparation method; however, the efficiency of the method is low. Therefore, the chemical method is a better choice with respect to cost and efficiency. Lignocellulosic scaffolds after delignification have been widely used in advanced functional materials, such as modified materials with excellent mechanical properties [16], low thermal conductivity [17,18], and thermal radiation cooling [19]. This suggests that delignification can expand the functional utilization of wood resources.

Bamboo is a lignocellulosic material composed of cellulose, hemicellulose, and lignin and is abundant, fast growing, sustainable, and renewable. Thus, bamboo fibres are considered potential raw resources for the fabrication of fibrous cellulose [20]. In bamboo composition, cellulose, hemicellulose, and lignin act as the skeleton, matrix, and encrusting materials, respectively [21]. Consequently, lignin removal causes lignocellulosic materials to become scaffolds that can be further decorated/grafted with different chemical functional groups, bringing novel and specific functionalities. Moreover, the mild conditions of delignification reactions (temperature; 80–120 °C and atmospheric pressure) do not impact the macrostructure substantially, preserving its original three-dimensional structure.

Although numerous studies have been conducted on the delignification of wood and bamboo and the functional transformation of treated materials, little information exists on changes in the material itself after delignification [22]. Alkali treatment is a common method for bamboo delignification. This study aimed to evaluate the microstructure, chemical change, and thermal degradation characteristics of bamboo delignified with sodium chlorite by comparing these characteristics before and after treatment. To the best of our knowledge, the microstructure, surface chemical composition, and pyrolysis behaviour of bamboo after sodium chlorite treatment have not been systematically studied yet. Hence, this study is expected to provide a reference basis for the functional value-added application of delignified bamboo.

## 2. Materials and Methods

### 2.1. Raw Materials

Five-year-old moso bamboo (*Phyllostachys pubescens* Mazel) was cut in Linan District, Zhejiang province. The samples were sawed 1.3 m from the base into 1 m long culms and divided into 20 mm wide and 0.5 mm thick strips. Sodium chlorite (2 wt.%) was obtained from Sinopharm Chemical Reagent Co., LTD (Huangpu, Shanghai, China), while acetic acid (AR grade) and ethanol were provided by Beijing Chemical Plant (DaXing, Beijing, China). Deionised water (DI) was made in the laboratory.

### 2.2. Delignification Treatment

First, 2000 mL of 2% sodium chlorite solution was prepared and the pH was adjusted to approximately 4.5 using acetic acid. Thereafter, the bamboo samples were completely soaked in sodium chlorite solution for 4 h at 50 °C. Subsequently, the samples were rinsed several times with DI, until the pH was neutral, to remove residual chemical substances. To better remove the residual reagent in the bamboo interior, the obtained samples were subjected to gradient dehydration using different ethanol concentrations (25, 50, 75, and 99.5 wt.%). The samples were immersed in each concentration of ethanol for 5 min in ascending order. Finally, all samples were washed with DI for 2 h to complete the bamboo delignification. All samples were stored in a room at 25 °C and 50% relative humidity for 15 days before the experimental study. The bamboo was categorised as: natural bamboo or NB (untreated bamboo) and delignified bamboo or DB (treated bamboo).

### 2.3. Characterization

The morphologies of NB and DB samples were measured using field-emission scanning electron microscopy (FE-SEM) (model SU8010, Hitachi, Japan). All samples were cut into tangential sections and coated with a gold layer.

For chemical analysis, Fourier transform infrared attenuated total internal reflectance (FTIR-ATR) spectra were obtained directly from the specimen surface using a Nicolet iS10 FTIR spectrometer (Thermo Scientific, Waltham, MA, USA) equipped with a diamond crystal ATR accessory (Smart iTX, Thermo Scientific, Waltham, MA, USA). For each measurement, 64 scans were conducted within 400–4000 cm^−^^1^ at 4 cm^−^^1^ resolution. Similarly, X-ray photoelectron spectroscopy (XPS) was performed at 150 W using a Thermo Scientific ESCALAB 250Xi spectrophotometer (Thermo Scientific, Waltham, MA, USA) equipped with a monochromatic Al Kα X-ray source (hv = 1486.6 eV), a 650 μm spot size, and pollutant carbon C1s = 284.8eV for charge correction. X-ray diffraction (XRD) data were obtained using a D8 ADVANCE X-ray instrument (Bruker, Karlsruhe, Germany) with Cu Kα radiation (wavelength, 1.5406 angstrom) at 40 kV, 40 mA, and a scan speed of 6°/min in the 2*θ* range of 5–50°. Pyrolysis characteristics were determined using a TG analyser (NETZSCH TG 209F1 Libra, Selb, Germany). A test temperature of 30–600 °C and linear heating rate of 10 °C/min^−1^ were employed under a N_2_ flow of 10 mL/min. Each sample weighed approximately 10 mg.

## 3. Results and Discussion

### 3.1. Surface Microstructure Morphology

**Figure 1:**
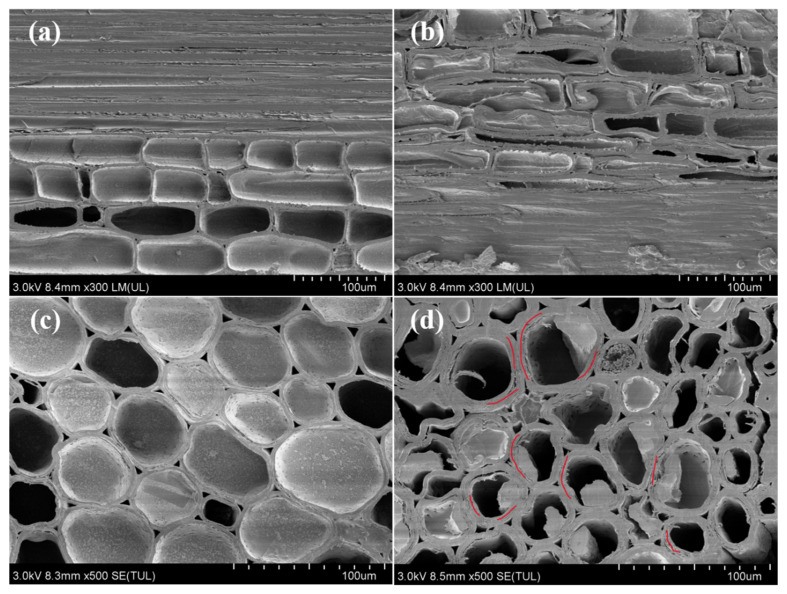
Scanning electron microscopy (SEM) images of (**a**,**c**) natural and (**b**,**d**) delignified bamboo. (**a**,**b**) Radial sections of natural bamboo (NB) and delignified bamboo (DB). (**c**,**d**) Cross sections of NB and DB.

As microstructural changes can affect the functional application of delignified bamboo, SEM was used for observation and analysis in the present study. The surface microstructure morphology of bamboo before and after sodium chlorite treatment is shown in Figure 1.

The SEM images of NB reveal that the radial section is laevigatus; moreover, the smooth surfaces of the cell wall are clearly visible (Figure 1a). The cross section shows the orderly arrangement of pores (Figure 1c). A comparison between Figure 1a,c and Figure 1b,d shows that the compact cell walls in the radial section and cross section became loose after delignification treatment. Additionally, many micron-scale pores and cellulose nanofibres were observed on the DB cell walls (red lines in Figure 1d). The present study’s findings confirm previous reports that delignification causes fibre cells to separate and jump out, resulting in a slight increase and decrease in the porosity and density of the treated bamboo, respectively [23]. Furthermore, the results confirm that alkali treatment causes a loss of bamboo matrix and the agglomeration of microfibres, which lead to size changes, surface roughness, cracking, and the loss of the mechanical strength of bamboo fibre [24].

### 3.2. Chemical Functional Groups

**Figure 2:**
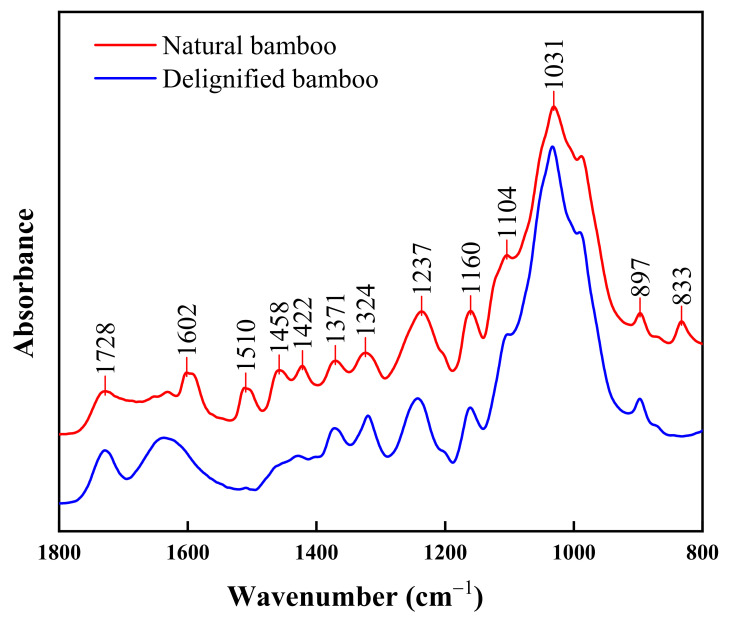
Fourier transform infrared (FTIR) spectra of NB and DB.

To further study which chemical composition changes in bamboo after sodium chlorite treatment led to microstructural changes, the results of the FTIR on DB and NB samples were compared and analysed. Figure 2 shows the FTIR spectra in the region ranging from 1800 to 800 cm^−1^, which reflects the entire molecule’s characteristics and is considered the fingerprint region of bamboo functional groups [22]. The FTIR spectra absorption peaks of the NB samples are defined in Table 1.

The regional range from 1800 to 800 cm^−1^ consisted of 13 absorption peaks (Figure 2). Seven peaks were attributed to the aromatic framework or main functional groups of lignin. The peaks at 1602, 1510, and 1422 cm^−1^ were attributed to the vibration contributions of C=C unsaturated linkages in the aromatic lignin skeleton, while the peak at 1458 cm^−1^ resulted from asymmetric bending in the CH_3_ of lignin. The absorption peak at 1237 cm^−1^ was attributed to the syringyl rings and C–O stretch in lignin and xylan. Additionally, the 1104 cm^−1^ peak value was due to the structural contributions of guaiacyl and syringyl in lignin. The curve of the DB in Figure 2 revealed that after sodium chlorite treatment, the intensity of the absorption peaks at 1602, 1510, 1458, 1422, 1237, and 1104 cm^−1^ weakened to various degrees or even disappeared. This finding signified that the lignin was substantially decomposed after the alkali treatment. Additionally, the peak at 833 cm^−1^ was attributed to C–H vibration in guaiacyl derivatives in lignin. The peak disappeared completely after delignification treatment, which further demonstrates that the lignin was decomposed.

In addition to lignin decomposition, the peak strengths (1728, 1371, 1324, and 897 cm^−1^) attributed to cellulose and hemicellulose showed only minor changes after the alkali treatment, in agreement with previous reports that chemical reactions between alkali and cellulose rarely occur [28]. However, the intensity of the peaks at 1160 and 1031 cm^−1^ slightly weakened, indicating that some hemicellulose may be degraded after treatment.

### 3.3. Chemical Composition

X-ray energy spectrum analysis (XPS) is a practical method for obtaining chemical and structural information on wood material surfaces [29]. Similar to wood, the chemical composition of bamboo comprises cellulose, hemicellulose, lignin, and small extract amounts, with carbon (C), hydrogen (H), and oxygen (O) as the main components. Therefore, the chemical properties of bamboo surfaces can be determined using XPS. In the present study, only the C and O elements in bamboo were assessed, as XPS cannot detect H elements. The main objects of XPS detection and analysis are 1s electrons in the inner shells of C and O atoms. Information on the chemical properties of the bamboo surfaces was obtained based on the C1s and O1s peak intensities and chemical shifts. The peaks of C_1_ and O_1_ were composed of components related to C and O functional groups in bamboo, respectively, and categorized as C_1_ (C–C, C–H), C_2_ (C–O), C_3_ (O–C–O, C=O), and C_4_ (O–C=O), and O_1_ (O–C=O) and O_2_ (C–O), according to the binding energy level. The C and O in different atomic binding states come from different sources [30,31]. The structural characteristics and chemical shifts of each are shown in Table 2.

**Figure 3:**
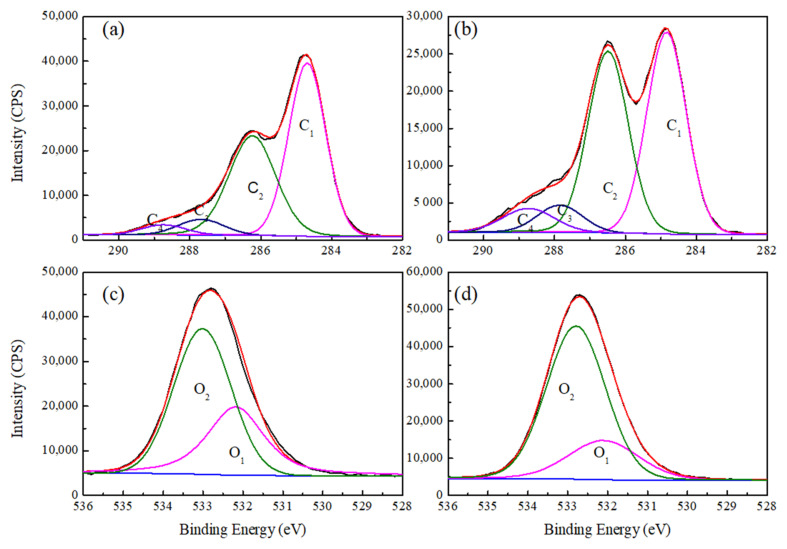
High-resolution X-ray photoelectron spectroscopy (XPS) spectra of (**a**,**b**) carbon and (**c**,**d**) oxygen peaks in (**a**,**c**) NB and (**b**,**d**) DB.

High-resolution C1s and O1s XPS spectra of natural bamboo and delignified bamboo were processed and are presented in Figure 3. Studies have shown that the degradation of cellulosic materials can be detected through a change in the oxygen-to-carbon (O/C) atomic ratio [22]. Quantitative measurements of O/C atomic ratios were calculated from Figure 3 using the total area of C and O peaks and their respective photoemission cross sections. The results are shown in Table 3.

In the C1s spectra, the peak components of C_1_ were primarily from lignin and extracts (C–C, C–H), while those of C_2_, C_3_, and C_4_ mainly originated from cellulose and hemicellulose (Table 2). After delignification treatment, the C_1_ content decreased from 51.71% to 44.88%, which showed that the lignin was effectively decomposed by sodium chlorite (Table 3). Additionally, the O/C atomic ratios given in Table 3 for NB and DB were 0.34 and 0.45, respectively. It has been reported that the O/C atomic ratios of cellulose, hemicellulose, lignin, and extracts (mainly Lipophilic compounds) are about 0.83, 0.8, 0.33, and 0.1 [29,35,36], respectively. High O/C ratios reflect high carbohydrate content, while low ratios indicate the presence of more lignin and extracts on the bamboo surface [37]. Therefore, the increased O/C atomic ratio in this study after delignification treatment further confirmed the degradation effect of sodium chlorite on lignin.

In the O1s spectra, the chemical components representing the O_1_ peak were lignin and extracts, and the O_2_ peak was associated with hemicelluloses and cellulose [38,39]. An analysis of the delignification treatment of the O1s spectra of bamboo samples revealed that O_1_ components decreased (from 37.48% to 23.96%) and O_2_ components increased (from 62.52% to 76.04%) (Table 3), which is similar to reports in previous studies [40].

### 3.4. Crystalline Structure

**Figure 4:**
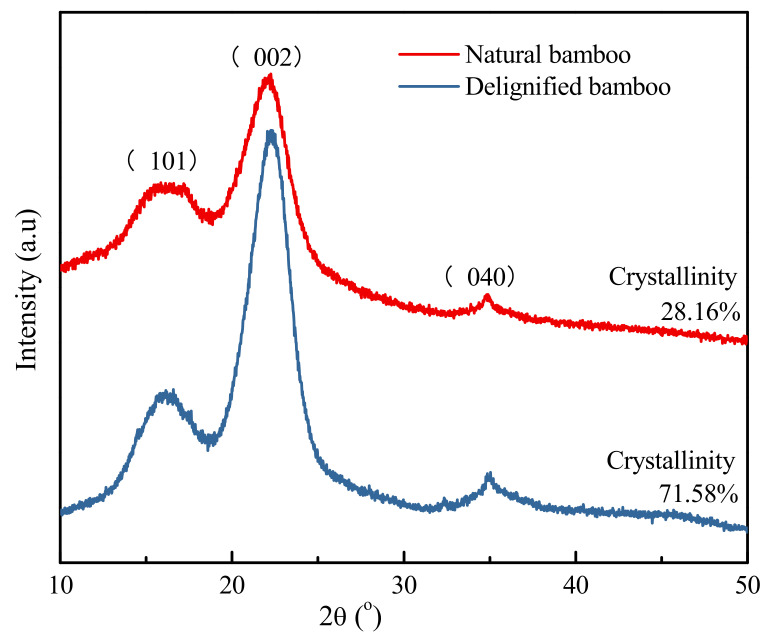
X-ray diffraction (XRD) patterns of NB and DB.

XRD can be used to investigate the supramolecular structure of biomass materials [41]. For example, using XRD techniques to analyse the crystallinity and crystal width provides insights into the effects of different treatments on the cellulose, hemicellulose, and lignin content of bamboo [42,43]. In the present study, XRD testing of NB and DB samples was conducted using an X-ray generator, and the results are illustrated in Figure 4.

Three typical peaks corresponding to the (040), (002), and (101) lattice planes of cellulose I were 34.66°, 22.26°, and 16.12°, respectively [44]. The intensity of the (040) peak was low; hence, the two reflections (101) and (002) were used to reflect the crystalline structure. Among them, the peak reflecting the crystal zone width was (002), and that reflecting the zone length was (040). The characteristic peaks of bamboo (101) and (002) corresponded to those of delignified bamboo, and their positions were almost unchanged, indicating that sodium chlorite treatment has almost no effect on the crystalline region of cellulose. However, the peak intensities of (101) and (002) of delignified bamboo were stronger than those of natural bamboo. It was observed from the calculation results that the crystallinity indices, after sodium chlorite treatment, were much higher than those of the natural bamboo (from 28.16% to 71.58%). This is because amorphous hemicellulose and lignin are selectively removed by sodium chlorite in the delignification process [45,46]. Simultaneously, the hydroxyl group of the amorphous microfibrils is exposed, forming hydrogen bonds with microfibrils on the surface of the crystallization region, thus improving the crystallinity.

### 3.5. Pyrolysis Properties

**Figure 5:**
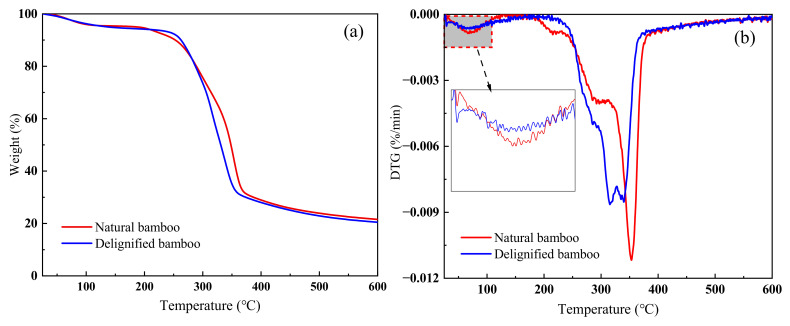
(**a**) Thermogravimetric analysis (TGA) and (**b**) derivative thermogravimetry (DTG) curves of NB and DB.

The pyrolysis of bamboo can be regarded as the superposition of the pyrolysis process of three main components (cellulose, hemicellulose, and lignin) [47]. Investigating the pyrolysis characteristics of bamboo before and after delignification treatment will help to better design and prepare functional bamboo composites by thermochemical conversion methods, such as gasification and pyrolysis [42]. Thermogravimetric analysis (TGA) was performed to study the alkali treatment effect on the pyrolysis characteristics of bamboo. The TGA and derivative thermogravimetry (DTG) curves of the samples are shown in Figure 5. Table 4 lists the corresponding data.

The difference between the NB and DB in the decomposition temperature was minor (Figure 5a). Notably, the pyrolysis process of sodium-chlorite-treated and -untreated bamboo was divided into two stages (Figure 5b). In the first stage, the temperature ranged from the initial temperature to approximately 140 °C. Small losses in sample mass were mainly caused by moisture loss, and the duration of when these losses occurred was identified as the water evaporation stage [48] (stage 1). A small weightlessness peak (fastest evaporation rate) appeared at 70.12 °C for the untreated bamboo and at 69.11 °C for the treated bamboo. The enlarged image in Figure 5b shows that the difference in the *R*_max_ values of these two temperature points is not large, and the slight difference can likely be attributed to the difference in initial conditions. In the natural state, the moisture content of different samples may be slightly different, which explains why the pyrolysis curves of the two samples in stage 1 are slightly different. In the second stage, which is the main reaction stage, the temperature ranged from the initial temperature to approximately 140 °C to 450 °C, and sample weightlessness was evident. In Table 4, we can see that the temperature of the treated bamboo samples was significantly lower than that of the untreated samples at the maximum thermal degradation rate (*R*_max_), indicating that sodium chlorite effectively removes lignin in bamboo fibre so that the remaining cellulose and hemicellulose are easily decomposed by heat. Additionally, the *R*_max_ value of the treated samples was significantly lower than that of the untreated bamboo (Figure 5b and Table 4), indicating that under similar pyrolysis conditions, lignin presence intensifies bamboo pyrolysis. Studies have shown that it is difficult to decompose lignin, that its weight loss occurs over a wide temperature range (160–900 °C), and that the amount of generated solid residue is very high (approximately 40 wt.%) [49]. Consequently, at the end of the experiment (the temperature reached 600 °C), the residual weight of the untreated bamboo samples was slightly larger than that of the treated bamboo, which was mainly caused by the presence of lignin.

## 4. Conclusions

Alkali treatment exhibited clear effects on the chemical composition of bamboo. Compared with the microstructure of untreated bamboo, the structure of bamboo treated with sodium chlorite was notably looser and coarser, with increased pores, decreased density, and the presence of fibres. The FTIR analysis results revealed that after bamboo treatment, the lignin-related peaks at 1602, 1510, 1458, 1422, 1237, 1104, and 833 cm^−1^ weakened or disappeared, meaning that the lignin was substantially decomposed. Similarly, the intensity of peaks at 1160 and 1031 cm^−1^ weakened slightly, indicating that some hemicellulose may be degraded after treatment. These changes directly result in the loose appearance of the microstructure. Additionally, the XPS analysis results revealed that the proportion of C_1_ and O_1_ attributed to lignin decreased from 51.71% to 44.88% and 37.48% to 23.96%, respectively, and that the O/C atomic ratio increased from 0.34 to 0.45, indicating that the surfaces of treated bamboo contain less lignin and fewer extracts. The XRD results showed that although the sodium chlorate treatment had little effect on the crystallization zone of bamboo cellulose, the crystallinity of treated bamboo increased significantly (from 28.16% to 71.58% at the end of the treatment). This was attributed to the fact that amorphous hemicellulose and lignin were removed during the treatment. Furthermore, the hydroxyl group of the amorphous microfibre was exposed and formed hydrogen bonds with the microfibre on the surface of the crystallization region, leading to increased crystallinity. TG analysis showed that bamboo pyrolysis before and after treatment could be divided into two stages: water evaporation and main decomposition. In the main decomposition stage, the maximum pyrolytic rate of treated bamboo was 315.62 °C, which was significantly lower than that of untreated bamboo, and the maximum pyrolysis rate was lower than that of untreated bamboo, indicating that after delignification, the intensity of bamboo pyrolysis decreases.

## Figures and Tables

**Table 1:** Assignment of FTIR spectra absorption peaks of natural bamboo [22,25,26,27].

Wavenumber (cm^−1^)	Functional Group	Assignment
1728	C=O	Non-conjugated C=O in hemicellulose (xylans)
1602	C=C	C=C unsaturated linkages, aromatic skeletal vibration in lignin
1510	C=C	Aromatic skeletal vibration (C=C) in lignin
1458	C–H, O–H	Asymmetric bending in CH_3_ (lignin)
1422	CH_2_	Aromatic skeletal vibrations (lignin) and C–H deformation in plane (cellulose)
1371	C–H	C–H deformation in cellulose and hemicellulose
1324	O–H	phenol group (cellulose)
1237	C–O	Syringyl ring and C–O stretch in lignin and xylan
1160	C–O–C	C–O–C vibration in cellulose and hemicellulose
1104	C–H	Guaiacyl and syringyl (lignin)
1031	C–O, C–H	C–O stretch in cellulose and hemicelluloseC–H stretch in lignin
897	C–H	C–H deformation in cellulose
833	C–H	C–H vibration in guaiacyl derivatives

**Table 2:** Classification of carbon (C) and oxygen (O) peak components of bamboo [32,33,34,35].

Element Component	Binding Energy (eV)	Binding Type	Main Resources
C_1_	284.5	C–C, C–H	Lignin and extracts
C_2_	285.5	C–O	Cellulose and hemicellulose
C_3_	286.5	O–C–O, C=O	Cellulose
C_4_	288.3	O–C=O	Hemicellulose and extracts
O_1_	532.8	O–C=O	Lignin
O_2_	534.1	C–O	Cellulose and hemicellulose

**Table 3:** Summary of XPS spectral parameters of NB and DB.

Samples	O/C Atomic Ratios	C (%)				O (%)	
		C_1_	C_2_	C_3_	C_4_	O_1_	O_2_
Natural bamboo	0.34	51.71	39.22	5.59	3.48	37.48	62.52
Delignified bamboo	0.45	44.88	41.65	6.73	6.73	23.96	76.04

**Table 4:** TGA data for NB and DB.

Sample	*T*_max_ (°C)		*R*_max_ (%/(°C)		Residues
	Stage 1	Stage 2	Stage 1	Stage 2	(wt.%)
Natural bamboo	70.12	353.78	8.39 × 10^−4^	111.71 × 10^−4^	21.68
Delignified bamboo	69.11	315.62	6.02 × 10^−4^	86.42 × 10^−4^	20.41

*T*_max_: temperature at the maximum weight-loss rate; *R*_max_: maximum decomposition rate.

## Data Availability

The data presented in this study are available on request from the corresponding author.
